# Ontogenetic differences in localization of glutamine transporter ApGLNT1 in the pea aphid demonstrate that mechanisms of host/symbiont integration are not similar in the maternal versus embryonic bacteriome

**DOI:** 10.1186/s13227-015-0038-y

**Published:** 2016-01-11

**Authors:** Hsiao-ling Lu, Daniel R. G. Price, Athula Wikramanayake, Chun-che Chang, Alex C. C. Wilson

**Affiliations:** Department of Biology, University of Miami, Coral Gables, FL 33146 USA; Department of Entomology, College of Bioresources and Agriculture, National Taiwan University, Taipei, Taiwan; Research Center for Developmental Biology and Regenerative Medicine, National Taiwan University, Taipei, Taiwan

**Keywords:** Host/symbiont developmental integration, Coevolution, Symbiosis, Amino acid transport, Holobiont, Bacteriome

## Abstract

**Background:**

Obligate intracellular symbionts of insects are metabolically and developmentally integrated with their hosts. Typically, reproduction fails in many insect nutritional endosymbioses when host insects are cured of their bacterial symbionts, and yet remarkably little is known about the processes that developmentally integrate host and symbiont. Here in the best studied insect obligate intracellular symbiosis, that of the pea aphid, *Acyrthosiphon pisum*, with the gammaproteobacterium *Buchnera aphidicola,* we tracked the expression and localization of amino acid transporter *ApGLNT1* gene products during asexual embryogenesis. Recently being characterized as a glutamine transporter, ApGLNT1 has been proposed to be a key regulator of amino acid biosynthesis in *A. pisum* bacteriocytes. To determine when this important mediator of the symbiosis becomes expressed in aphid embryonic bacteriocytes, we applied whole-mount in situ hybridization and fluorescent immunostaining with a specific anti-ApGLNT1 antibody to detect the temporal and spatial expression of *ApGLNT1* gene products during asexual embryogenesis.

**Results:**

During embryogenesis, *ApGLNT1* mRNA and protein localize to the follicular epithelium that surrounds parthenogenetic viviparous embryos, where we speculate that it functions to supply developing embryos with glutamine from maternal hemolymph. Unexpectedly, in the embryonic bacteriome ApGLNT1 protein does not localize to the membrane of bacteriocytes, a pattern that leads us to conclude that the regulation of amino acid metabolism in the embryonic bacteriome mechanistically differs from that in the maternal bacteriome. Paralleling our earlier report of punctate cytoplasmic localization of ApGLNT1 in maternal bacteriocytes, we find ApGLNT1 protein localizing as cytoplasmic puncta throughout development in association with *Buchnera*.

**Conclusions:**

Our work that documents ontogenetic shifts in the localization of ApGLNT1 protein in the host bacteriome demonstrates that maternal and embryonic bacteriomes are not equivalent. Significantly, the persistent punctate cytoplasmic localization of ApGLNT1 in association with *Buchnera* in embryos prior to bacteriocyte formation and later in both embryonic and maternal bacteriomes suggests that ApGLNT1 plays multiple roles in this symbiosis, roles that include amino acid transport and possibly nutrient sensing.

**Electronic supplementary material:**

The online version of this article (doi:10.1186/s13227-015-0038-y) contains supplementary material, which is available to authorized users.

## Background

Vertically transmitted symbionts are developmentally and metabolically integrated with their hosts to generate the holobiont (host + symbiotic microorganism) [1]. In most systems, remarkably little is known about the mechanisms mediating holobiont integration. Here we address this knowledge deficit by studying integration of the pea aphid, *Acyrthosiphon pisum*, and its endosymbiont, *Buchnera aphidicola*, during parthenogenetic viviparous embryogenesis using *A. pisum* glutamine transporter, ApGLNT1 [2].

*Buchnera* are the maternally inherited obligately intracellular nutritional symbionts of almost all extant aphids [3, 4]. In adult females, *Buchnera* are contained in a maternal bacteriome, which is an organ-like structure that comprises an aggregation of bacteriocytes and sheath cells. Each bacteriocyte houses thousands of individual *Buchnera* enveloped in host-derived symbiosomal membranes, while sheath cells that are located at the periphery of bacteriocytes and sometimes contain secondary bacterial symbionts do not contain *Buchnera* [5–7]. The transovarial inheritance and developmental integration of *Buchnera* can be divided into three phases: transmission, cellularization, and maturation. Occurring early in the development of blastoderm embryos, transmission involves exocytosis of *Buchnera* from maternal bacteriocytes—a process that results in release of *Buchnera* from maternal bacteriocytes and loss of the symbiosomal membrane. Following release, naked *Buchnera* move through the maternal extracellular space via cytoplasmic extensions that extend from maternal bacteriocytes to the inner space of the blastula [8]. Prior to gastrulation, *Buchnera* invade blastula embryos between the posterior enlarged follicle cells [9] resulting in *Buchnera* cells reacquiring their host-derived symbiosomal membrane [8]. After invasion, *Buchnera* cells aggregate in the posterior egg chamber with uncellularized host nuclei [9, 10]. Cellularization—the second phase of developmental integration—follows embryo gastrulation when the *Buchnera* population compartmentalizes into individual bacteriocytes and proliferates [10]. Maturation—the final phase of developmental integration—occurs during late embryogenesis (stages 16–19) after katatrepsis (also known as “embryo flip”, the second event of the blastokinesis that is peculiar to hemimetabolous insects). During maturation, uninucleate bacteriocytes and the intervening sheath cells located around the bacteriocytes form the dorsally localized bacteriome [8–10].

*Buchnera*, like other obligate intracellular symbionts of insects that feed on plant sap, provide their hosts with essential amino acids that are found only at very low concentrations in plant sap [11]. Amino acid biosynthesis in the *A. pisum*/*Buchnera* holobiont occurs in bacteriocytes. Transport of amino acids from aphid hemolymph into bacteriocytes across the symbiosomal membrane and the inner and outer membranes of *Buchnera* and then back out to aphid hemolymph is central to symbiotic function. Recently, one amino acid transporter, ApGLNT1, has been proposed to regulate amino acid biosynthesis in bacteriocytes, thus ensuring that amino acid supply meets host demand [2].

Amino acid transporter ApGLNT1 is part of an arthropod expanded clade of eukaryotic-specific amino acid/auxin permease (AAAP) family transporters (Transporter Classification # 2.A.18) and closely related to the mammalian solute carrier 36 (SLC36) family [12]. Notably, the predicted membrane topology and proton-dependent uptake characteristics of ApGLNT1 are similar to those of the mammalian SLC36 family [13, 14]. Being highly expressed in *A. pisum* gut and bacteriocyte tissue, ApGLNT1 has remarkably narrow substrate selectivity with high glutamine and low arginine transport function [2, 12, 13]. While the arginine transport capacity of ApGLNT1 is low, its arginine binding affinity is high and, thus, arginine functions as a competitive inhibitor of glutamine transport [2]. Coupling the transport capacity of ApGLNT1 with its localization to the membrane of adult bacteriocytes led Price et al. [2] to propose that ApGLNT1 is the key regulator of amino acid biosynthesis in the *A. pisum*/*Buchnera* holobiont.

Amino acid transporters play an important role in amino acid transport at the symbiotic interface in all biological systems that include an obligate intracellular symbiont. One group of insects that critically depend on vertically transmitted, obligate intracellular symbionts includes plant sap-feeding insects of the suborder Sternorrhyncha. Remarkably, in earlier work we have found that nutrient amino acid transporter gene families are expanded in Sternorrhyncha insects that include the whitefly *Bemisia tabaci*, the potato psyllid *Bactericera cockerelli*, the citrus mealybug *Planococcus citri*, and the pea aphid *Acyrthosiphon pisum* [15, 16]. In all these insects, a subset of amino acid transporter paralogs shows patterns of bacteriocyte-biased gene expression suggesting that neofunctionalization or subfunctionalization of duplicated paralogs facilitates host/symbiont metabolic integration [12, 15]. While the genomic basis of host/symbiont metabolic integration is increasingly well understood [17], the developmental basis of host/symbiont integration has been less studied. That said, recent work in the hemipteran insect *Nysius plebeius* has started to reveal the molecular and developmental mechanisms that drive bacteriocyte differentiation [18]. Here we study the developmental integration of the nutritional symbiont *Buchnera* by extending the work of Price et al. [2] to investigate the expression and localization of *ApGLNT1* gene products through viviparous development in the pea aphid. We show that RNA expression and protein localization of *ApGLNT1* are different in the maternal versus embryonic bacteriome leading us to conclude that host/symbiont integration and regulation are not ontogenetically constant.

## Methods

### Aphids

*A. pisum* line LSR1 [19] was maintained on *Vicia faba* and incubated at 20 °C under a 16-h light/8-h dark cycle. Oocytes, embryos, and bacteriocytes were dissected for whole-mount RNA in situ hybridization and fluorescent immunostaining from wingless adult females in phosphate-buffered saline (PBS; 10 mM phosphate buffer, 154 mM NaCl, pH 7.4; Sigma-Aldrich). Oogenesis and embryogenesis staging was according to Miura et al. [9] with germ cell locations according to Chang et al. [20].

### Validation of Anti-ApGLNT1 antibody against ApGLNT1 protein

Here we use the monospecific anti-ApGLNT1 antibody purified from rabbit sera as described by Price et al. [2]. This antibody was raised against amino acids 28–40 of ApGLNT1 (LDNNKRGSIRTDV). Earlier validation by Price et al. [2] of this antibody included preadsorbed controls run in parallel with all experiments. Here we further validate the anti-ApGLNT1 antibody by Western blot. Briefly, a full-length coding sequence for *ApGLNT1* [GenBank: NM_001246261.1] was amplified from whole adult female *A. pisum* cDNA using Phusion proof-reading polymerase (Finnzymes). The primers contained a 5′ optimized Kozak initiation sequence for efficient translation in yeast [21], a 5′ NotI site, and a 3′ BamHI site. The forward primer sequence is 5′-AA**GCGGCCGC**ATAATGGCGCATCATT-3′ and the reverse primer sequence is 5′-TT**GGATCC**TCACTGATTGAACGTTAC-3′; restriction enzyme sites are shown in bold. The amplified *ApGLNT1* coding sequence was digested with NotI and BamHI and cloned into the respective sites of the yeast shuttle vector pDR195 [22]. The *ApGLNT1* expression construct was fully sequenced and used to transform *Saccharomyces cerevisiae* strain 22Δ8AA [23] by the lithium acetate/PEG method [24]. Transformants were selected on synthetic complete (SC) media, pH 5.6 (0.17 % yeast nitrogen base, 2 % maltose, 1 % agar supplemented with uracil drop-out mix) at 30 °C for 3–4 days. Controls were run in parallel and consisted of cells transformed with empty pDR195 expression vector (negative control).

Total membrane fractions were isolated from 22Δ8AA cells as described by Sauer and Stolz [25]. Membrane proteins were resolved by SDS-PAGE on a 12.5 % polyacrylamide gel containing 0.1 % SDS in a discontinuous pH system [26]. Separated proteins were transferred to nitrocellulose membranes by electroblotting in Bjerrun and Schafer-Nielsen buffer with SDS (48 mM Tris, 39 mM glycine, 20 % methanol, 0.0375 % SDS, pH 8.3). Transferred proteins were probed with rabbit anti-ApGLNT1 antibody [2] at 1:1000 dilution, followed by secondary IRDye 800CW Goat anti-rabbit IgG (H + L) (LI-COR) at 1:10,000 dilution. Bound antibodies were visualized using a LI-COR Odyssey infrared imaging system model 9120.

Western blot analysis revealed that the anti-ApGLNT1 polyclonal antibody recognizes a single major band of recombinant ApGLNT1 protein that was extracted from the membrane fractions of the yeast *S. cerevisiae* expressing ApGLNT1 (Additional file 1: Figure S1). These data validate that the anti-ApGLNT1 antibody is specific to endogenous ApGLNT1 as described by Price et al. [2].

### Whole-mount RNA in situ hybridization

Riboprobes were prepared from a 509 bp fragment of the *ApGLNT1* coding sequence in a region present in all four *ApGLNT1* alternative splice variants present in the NCBI database, i.e., 524–1032 of NM_001246261, 1182–1690 of XM_008184610, 1176–1684 of XM_008184611, and 543–1051 of XM_008184612. *ApGLNT1* was amplified from plasmids containing full-length *ApGLNT1* cDNA [2] using forward 5′-CCTTCCAAAAATTTTCCGGT-3′ and reverse 5′-GAGGAAGCCAAACATTCCAA-3′ primers. Amplification conditions consisted of an initial denaturation at 94 °C for 30 s, followed by 35 cycles at 94 °C for 10 s, 50 °C for 30 s, 72 °C for 1 min, and a final extension at 72 °C for 5 min. Amplicons were subcloned into pGEM-T vector (Promega). Finally, DIG-labeled sense and antisense *ApGLNT1* riboprobes were in vitro transcribed from linearized plasmids containing verified *ApGLNT1* sequences using SP6 RNA polymerase (New England Biolabs) and T7 RNA polymerase (New England Biolabs), respectively.

Bacteriocytes and ovaries containing developing oocytes and embryos were fixed in 3.8 % formaldehyde (VWR) in PBS at 4 °C overnight. The working concentration of each probe, including sense and anti-sense strands, was 3.0 ng/µl. Other steps follow the protocol of Chang et al. [27]. Probe hybridization was performed at 68 °C and nitroblue tetrazolium (NBT)/5-bromo-4-chloro-3-indolyl phosphate (BCIP) (Roche) was applied as the substrate for signal development. The substrate reaction was terminated concurrently for experimental and control treatments based on the relative intensity of signal in the follicle cells in the anti-sense riboprobe (experimental) and sense riboprobe (control) treatments. Following signal development, samples were mounted in 70 % glycerol (Sigma-Aldrich) in PBS and photographed with a Zeiss Axiovert 200 microscope connected to a Zeiss AxioCam ICc1 camera. The experiment was performed five times for embryos and three times for bacteriocytes.

### Fluorescent immunostaining in oocytes and embryos

Dissected ovaries that included developing oocytes and developing embryos were fixed in 4 % paraformaldehyde (Thermo Scientific) in PBS for 20 min. Immunostaining followed the protocol of Chang et al. [28] but omitted the H_2_O_2_ treatment step. Ovaries were incubated in a 1:20 dilution of rabbit anti-ApGLNT1 polyclonal antibody [2] at 4 °C overnight, and the rabbit IgGs were detected with Alexa Fluor 633-conjugated goat anti-rabbit IgG (H + L) antibody (Invitrogen) at 1:500 dilution for 2–4 h at room temperature. Controls included a secondary antibody only negative control and an ApVasa1 (ApVas)-positive control [29]. Nuclei and F-actin were stained with 2 μg/ml of 4′,6-diamidino-2-phenylindole (DAPI) (Sigma-Aldrich) and 0.5 μg/ml of phalloidin–tetramethylrhodamine B isothiocyanate (phalloidin–TRITC) (Sigma-Aldrich) at room temperature for 1 h. Samples were then mounted in 70 % glycerol (Sigma-Aldrich) in PBS at 4 °C overnight. Images were acquired using a Leica TCS SP5 laser scanning confocal microscope in the University of Miami, Department of Biology Microscopy Core Facility. Control treatments were run in parallel. The experiment was performed five times.

## Results

### *ApGLNT1* mRNA is expressed in the maternal follicular epithelium external to embryos and in sheath cells of the post-embryonic bacteriome

**Fig. 1:**
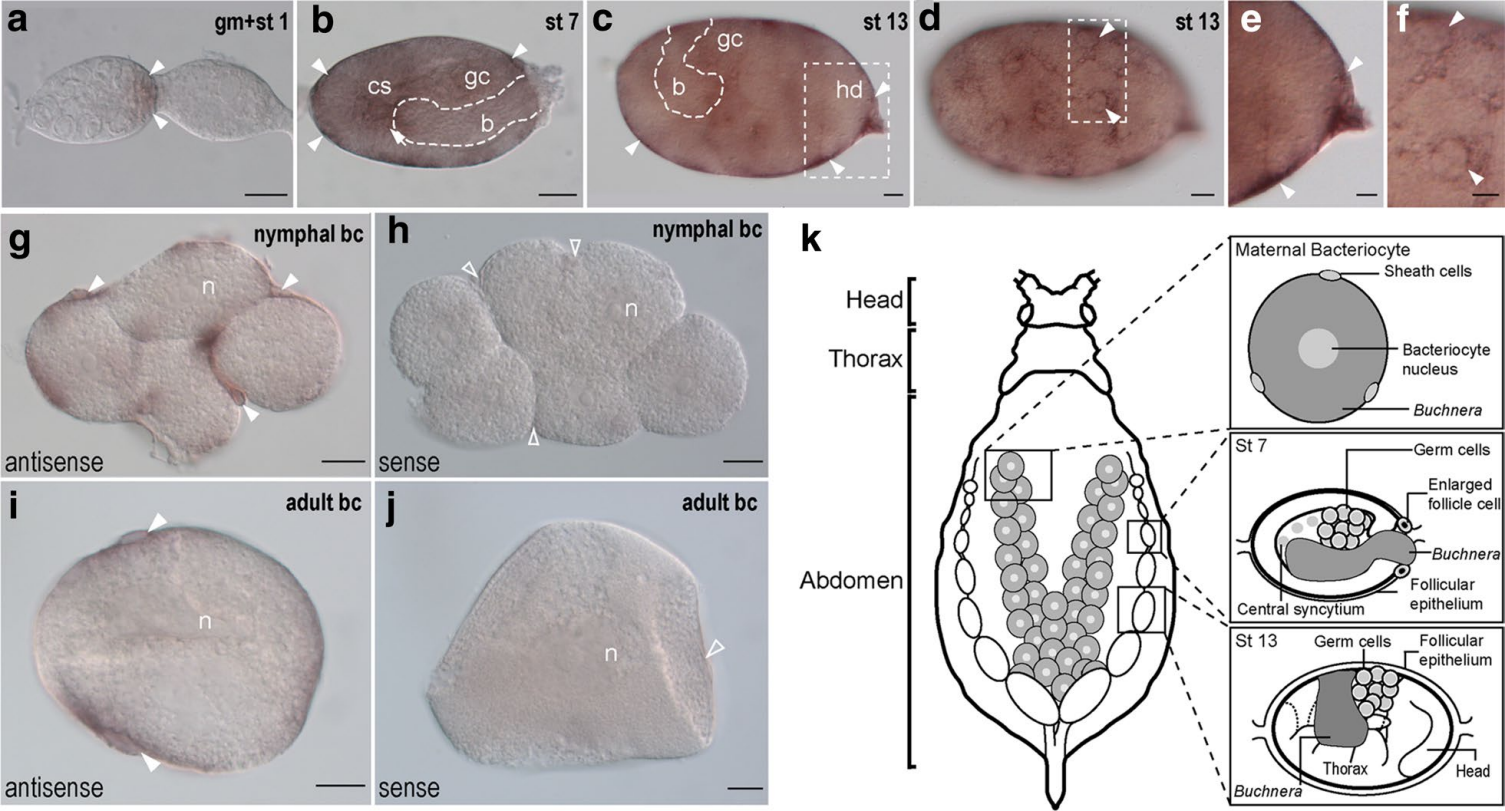
Developmental expression of *ApGLNT1* mRNA in the pea aphid. **a**–**f** Germaria and stage 1 oocyte (**a**), stage 7 (**b**) and stage 13 (**c**–**f**) embryos of the asexual pea aphid were hybridized with *ApGLNT1* antisense riboprobes, and the transcripts were visualized with a color substrate NBT/BCIP. **c** and **d** are the same sample but at different focal planes. **e** and **f** are the *insets* of **c** and **d**, respectively. The anterior of germaria is to the *left*. The head (hd) of germband is indicated in the posterior of the egg chamber to the *right*. The *arrow* indicates the antisense riboprobe-positive signal in the central syncytium (cs) and *arrowheads* indicate signals in the follicle cells in the periphery of egg chamber. **g**–**j** Maternal bacteriome dissected from 24–48-h-old nymphs (**g**, **h**) and adult (**i**, **j**) hybridized with antisense and sense riboprobes. The expression of *ApGLNT1* is indicated with closed *arrowheads* located in the sheath cells. The *open arrowheads* in the sense group indicate the regions where *ApGLNT1* expression was detected when tissues were probed with anti-sense probes. **k** Illustration displaying presented bacteriocytes and developmental stages of embryos used for whole-mount in situ hybridization. *Scale bars*: 20 μm. *b* endosymbiotic bacteria *Buchnera*, *bc* bacteriocyte, *cs* central syncytium, *gc* germ cells, *gm* germarium, *hd* head, *n* bacteriocyte nucleus, *st* stage

During early development (stages 0–4), *ApGLNT1* transcripts are detected in the follicle cells between the germarium that is composed of nurse cells and presumptive oocytes and the first egg chamber (Fig. 1a; Additional file 2: Figure S2a, b). From stage 4 onward, *ApGLNT1* mRNA is preferentially expressed in the follicular epithelium surrounding the egg chambers (Fig. 1b–f). Notably, we found that *ApGLNT1* is enriched in a region around the follicular cell nuclei in the follicular epithelium (Fig. 1d, f; Additional file 2: Figure S2c–i). Such epithelial localization of *ApGLNT1* transcripts continues through katatrepsis until the end of development (stage 19). While the most obvious and persistent expression of *ApGLNT1* occurs in the follicular epithelium, we also detected expression of *ApGLNT1* in the central syncytium concurrent with symbiont invasion (stage 7; Fig. 1b). However, we did not find *ApGLNT1*-specific signals in the location of *Buchnera*, nor did we find it in the embryonic head, or the embryonic alimentary canal.

In addition to examining *ApGLNT1* expression through development, we studied the subcellular localization of *ApGLNT1* transcripts in the maternal bacteriome of nymphal and adult asexual females. In the maternal bacteriome of nymphal and adult asexual females, we detected *ApGLNT1* transcripts in sheath cells but not within the cytoplasm of the bacteriocytes (Fig. 1g–j).

The patterns described above were repeatable across all five embryonic and all three bacteriocyte experimental replicates.

### ApGLNT1 protein is localized to the maternal follicular epithelium and embryonic sheath cells

**Fig. 2:**
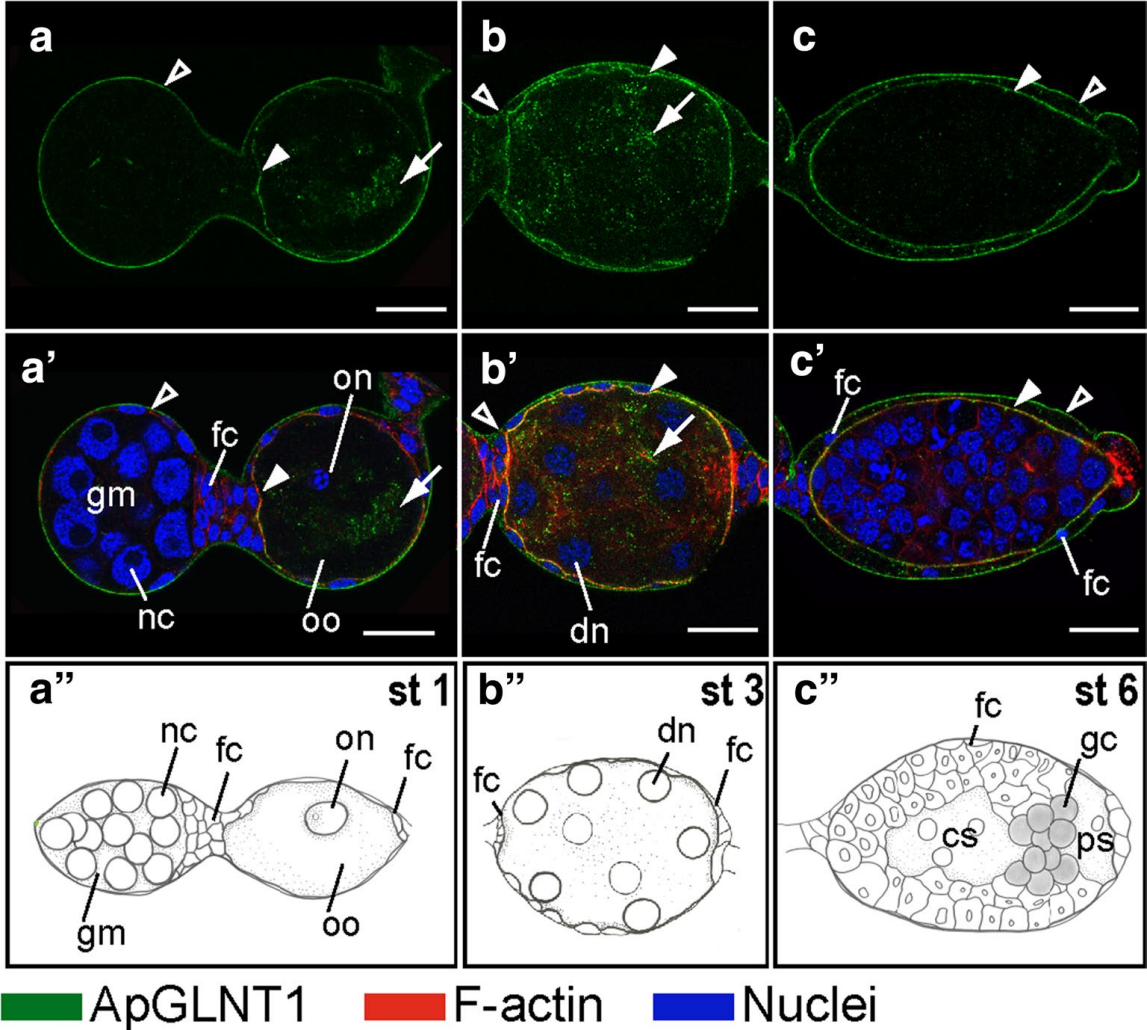
Developmental expression of ApGLNT1 in early developing embryos before symbiont transmission. Staining with ApGLNT1 antibody was performed on dissected asexual embryos within the ovarioles. All embryos are displayed with anterior of the germaria to the *left.* Signals representing ApGLNT1 immunoactivity, F-actin to the cell cortex, and nuclei are marked with *color keys* at the *bottom* of the figure. Confocal images of **a**–**c** show ApGLNT1 antibody staining results. Confocal images of **a**′-**c**′ show merged results from ApGLNT1 antibody, nuclei, and F-actin staining. Illustrations of **a**″–**c**″ display the embryonic characteristics corresponding to the presented developmental stages. ApGLNT1 signal in the outer layer surrounding the whole ovarioles is indicated with *open arrowheads*, and the signal in the inner layer surrounding the developing oocyte and embryos is indicated with *closed arrowhead*. **a**, **a**′, **a**″ Germaria and developing oocyte (stage 1 of development). **a**, **a**′ ApGLNT1 signals are detected in the membrane of follicular epithelium (*closed and open arrowheads*) and in the cytoplasm of the oocyte (*arrow*). **b**, **b**′, **b**″ Embryo undergoing synchronous, syncytial nuclear division with eight nuclei (stage 3 of development). **b**, **b**′ ApGLNT1 signals are localized in the membrane of follicular epithelium (*closed and open arrowheads*) and in the cytoplasm of the syncytial embryos (*arrow*). **c**, **c**′, **c**″ Embryo with newly segregated germ cells (stage 6 of development). **c**, **c**′ The membrane signals in the follicular epithelium remain detectable in the periphery of the embryos (*closed and open arrowheads*). *Scale bars*: 20 μm. *cs* central syncytium, *dn* dividing nuclei, *fc* follicle cells, *gc* germ cells, *gm* germarium, *nc* nurse cells, *on* oocyte nucleus, *oo* oocyte, *ps* posterior syncytium, *st* stage

**Fig. 3:**
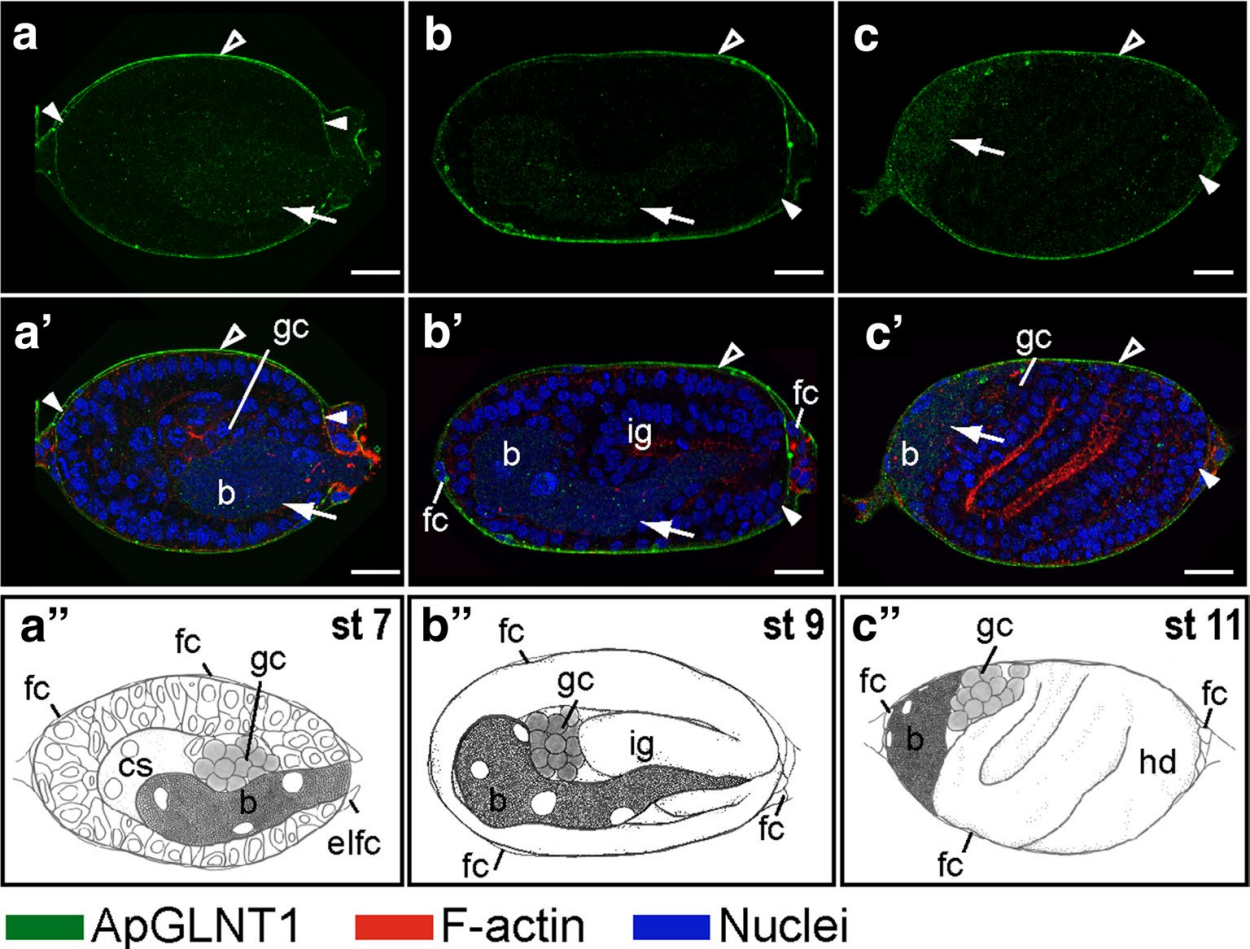
Developmental expression of ApGLNT1 in the embryos when the maternal symbiont undergoes transmission. Signals representing ApGLNT1 immunoactivity, F-actin to the cell cortex, and nuclei are marked with *color keys* at the *bottom* of the figure. All embryos are displayed with anterior of the germaria to the *left*. Confocal images of **a**–**c** show ApGLNT1 antibody staining results. Confocal images of **a**′–**c**′ show merged results from ApGLNT1 antibody, nuclei, and F-actin staining. Illustrations of **a**″–**c**″ display the embryonic characteristics corresponding to the presented developmental stages. ApGLNT1 signal in the outer layer surrounding the whole ovarioles is indicated with *open arrowhead*, and signal in the inner layer surrounding the developing embryos is indicated with *closed arrowhead*. The punctate signal of ApGLNT1 localized in the endosymbiotic bacteria region is indicated with an *arrow*. **a**, **a**′, **a**″ Embryo incorporating the maternal endosymbiotic bacteria (stage 7 of development). **a**, **a**′ ApGLNT1 signals are detected in the membranes of the follicular epithelium (*closed and open arrowheads*) and in the invading endosymbiotic bacteria (*arrow*). **b**, **b**′, **b**″ Invaginating embryo (stage 9 of development). **b**, **b**′ Similar to stage 7 embryo, ApGLNT1 signals are localized in the membranes of follicular epithelium (*closed and open arrowheads*) and in the invading endosymbiotic bacteria (*arrow*). **c**, **c**′, **c**″ S-shaped embryo (stage 11 of development). **c**, **c**′ The signal of follicular epithelium remains detectable in the periphery of the embryos (*closed and open arrowheads*) and punctate signals in the region of endosymbiotic bacteria become obvious (*arrow*). *Scale bars*: 20 μm. *b* endosymbiotic bacteria *Buchnera*, *cs* central syncytium, *elfc* enlarged follicle cells, *fc* follicle cells, *gc* germ cells, *hd* head, *ig* invaginating germband, *st* stage

ApGLNT1 protein localization is spatially coincident with *ApGLNT1* transcripts in the maternal follicular epithelium. We observed two layers of contiguous punctate signals: one along the outer edge of the maternal epithelium and the other surrounding the embryos (Fig. 2a–c, a′–c′). As embryos develop and grow the maternal epithelium flattens, so that by around stage 7 it is difficult to discern the double layer of ApGLNT1 signal using confocal imaging (Fig. 3a, a′).

**Fig. 4:**
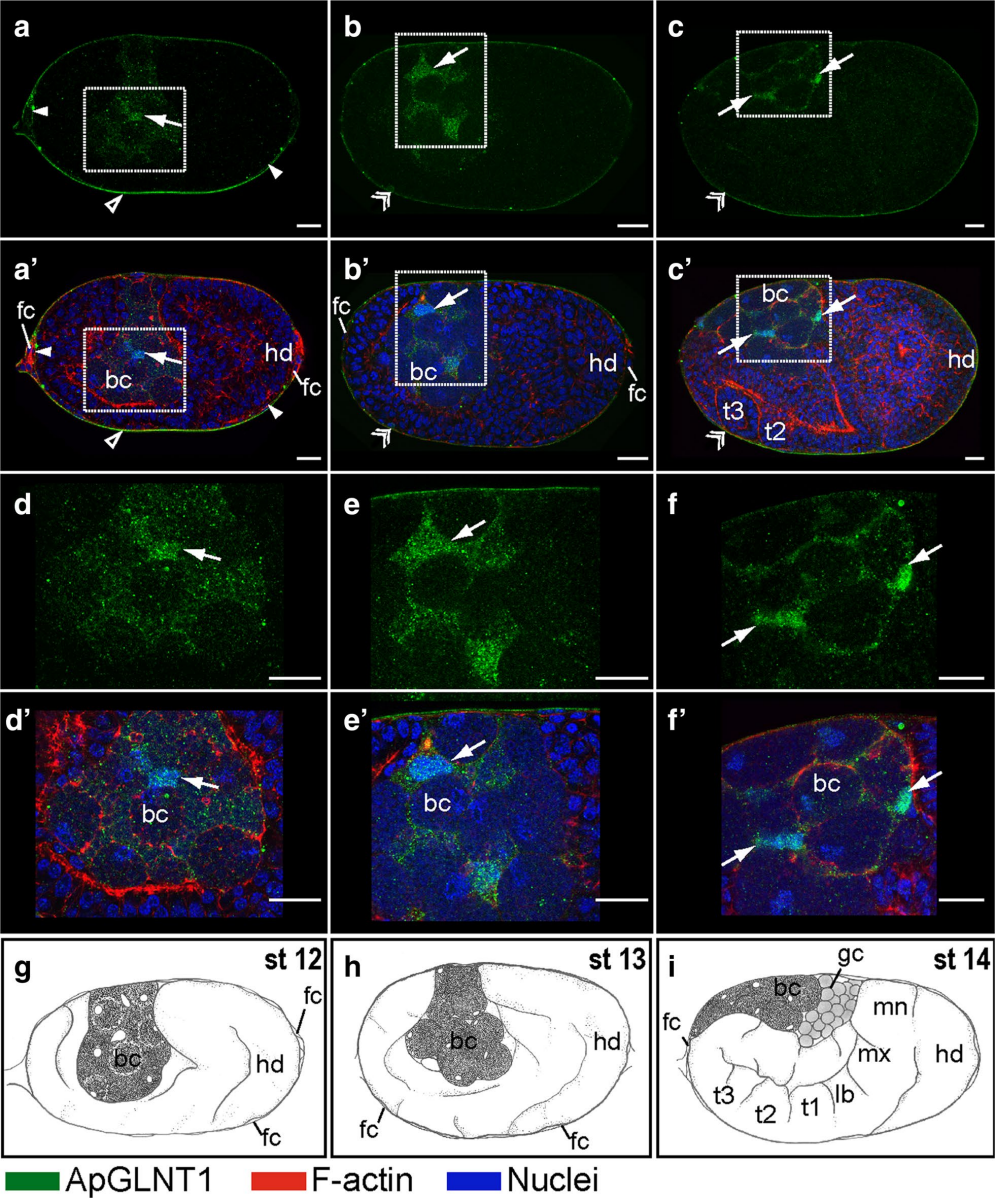
Developmental expression of ApGLNT1 in the embryos when bacteriocyte undergoes cellularization. Signals representing ApGLNT1 immunoactivity, F-actin to the cell cortex, and nuclei are marked with *color keys* at the *bottom* of the figure. All embryos are displayed with anterior of the germaria to the *left*. Confocal images of **a**–**c**; **d**–**f** show ApGLNT1 antibody staining results. Confocal images of **a**′–**c**′; **d**′–**f**′ show merged results from ApGLNT1 antibody, nuclei, and F-actin staining. **d**–**f** and **d**′–**f**′ are the insets shown in **a**–**c** and **a**′–**c**′, respectively. Illustrations of **g**–**i ** display the embryonic characteristics corresponding to the presented developmental stages. ApGLNT1 signal in the outer layer surrounding the whole ovarioles is indicated with an *open arrowhead*, the inner layer surrounding the embryos is indicated with a *closed arrowhead*, and when two layer signals are indiscernible indicated with *double arrowheads*. ApGLNT1 signal located within the bacteria region is indicated with *arrows*. **a**, **a**′, **d**, **d**′, **g** Twisting embryo (stage 12 of development). **a**, **a**′, **d**, **d**′ ApGLNT1 signals are detected in the membranes of follicular epithelium (*closed and open arrowheads*) and in the periphery of cellularized bacteriocytes (*arrow*). **b**, **b**′, **e**, **e**′, **h** Embryo undergoing limb bud formation (stage 13 of development). **b**, **b**′, **e**, **e**′ Follicular epithelium signal can be detected (*double arrowhead*). ApGLNT1 signal in the periphery of the cellularized bacteriocytes becomes more condensed (*arrow*). **c**, **c**′, **f**, **f**′, **i** Extension of the germband (stage 14 of development). **c**, **c**′, **f**, **f**′ The follicular epithelium signal remains detectable in the periphery of the egg chamber (*double arrowhead*). ApGLNT1 signal in the periphery of the cellularized bacteriocytes becomes more restricted (*arrows*). *Scale bars*: 20 μm. *bc* bacteriocyte, *fc* follicle cells, *gc* germ cells, *hd* head, *lb* labial segment, *mn* mandible segment, *mx* maxilla segment, *st* stage, *t1*–*t3* the three thoracic segments

At stage 7, concomitant with *Buchnera* invasion weak punctate signals of ApGLNT1 can be detected in the bacterial region (arrow in Fig. 3a, a′). The intensity of these puncta increases during early gastrulation (Fig. 3b, b′, c, c′). From stage 7 to stage 11, the punctate staining of ApGLNT1 is enriched in the region occupied by *Buchnera* (Fig. 3a–c, a′–c′). However, starting at stage 12, during the process of bacteriocyte cellularization, ApGLNT1 puncta are enriched at the periphery of bacteriocytes, eventually localizing to what we interpret to be the interstitial region between bacteriocytes (in mature bacteriomes this interstitial region is occupied by sheath cells) (Fig. 4a, a′, d, d′). As development proceeds through stages 12 and 13, the intensity of the ApGLNT1-positive signal in interstitial regions around bacteriocytes increases (Fig. 4b, b′, e, e′). Notably, during germband formation (stage 13) and limb bud formation (stage 14), ApGLNT1-positive signals are always found in the nucleus and cytoplasm of putative sheath cells (arrow in Fig. 4c, c′, f, f′, and for more detail see Additional file 3: Movie S1).

**Fig. 5:**
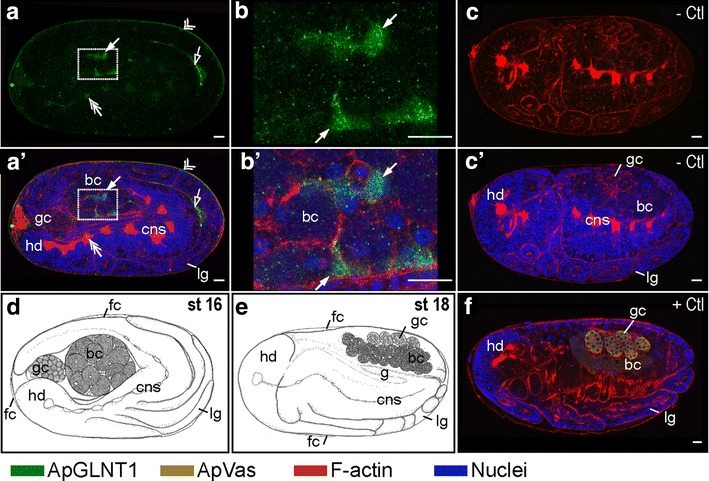
Developmental expression of ApGLNT1 in post-katatrepsis embryos when bacteriome undergoes maturation. Signals representing ApGLNT1 and ApVas immunoactivity, F-actin, and nuclei are marked with *color keys* below the figure. All embryos are displayed with anterior of the germaria to the *left*. Confocal images of **a** and **b** show ApGLNT1 antibody staining results. **a**′ and **b**′ show merged results from ApGLNT1 antibody, nuclei, and F-actin staining. **b** and **b**′ are the *insets* of **a** and **a**′, respectively. **c** and **c**′ are the negative controls. Illustrations of **d** and **e** display the embryonic characteristics corresponding to the presented developmental stages. **f** ApVas-positive control of staining. **a**, **a**′, **b**, **b**′ Embryo after flip (stage 16). **a**, **a**′, **b**, **b**′ The ApGLNT1 signal can be detected in the follicular epithelium (*double arrowhead*), in the presumptive bacterial sheath cells (*arrow*) of the bacteriome, and in the central nervous system (*double arrow*). An ApGLNT1-positive signal can be detected in the space of germband and limb bud (*open arrow*). **c**, **c**′ Embryo undergoes retraction (stage 17). **c** Embryo stained with only secondary antibody and merged with the F-actin staining and **c**′ merged with nucleus staining. In the absence of anti-ApGLNT1 antibodies, no immunostaining signal is detected. **f** Stage 18 embryo stained with ApVas antibody as a positive control. Signal specifically appears in the germ cells. *Scale bars*: 20 μm. *bc* bacteriocyte, *cns* central nervous system, *fc* follicle cells, *g* gut, *gc* germ cells, *hd* head, *lg* legs, *st* stage, − Ctl: negative control, + Ctl: positive control

Following katatrepsis (stage 16 onward) (Fig. 5a, a′, b, b′), the bacteriome undergoes maturation [8]. During maturation, ApGLNT1-positive signals are found in the maternal follicular epithelium (double arrowhead in Fig. 5a, a′) and the sheath cell precursors (arrow in Fig. 5b, b′). In addition, ApGLNT1 signal can be observed in the central nervous system (double arrow in Fig. 5a, a′) and at limb/abdomen junctions (open arrow in Fig. 5a, a′). We are confident that all signals were generated by the anti-ApGLNT1 antibody because the negative controls were always almost completely devoid of signal (Fig. 5c, c′).

The patterns described above were repeatable across all five replicate experiments. To validate immunostaining procedures and the efficiency of antibody penetration, all experiments included an ApVas-positive control. ApVas is a marker that specifically stains pea aphid primordial germ cells, and the localization of ApVas protein during development is well characterized (Fig. 5f) [29].

## Discussion

### Protein localization patterns in the maternal and embryonic bacteriomes are not equivalent

In the adult maternal *A. pisum* bacteriome, amino acid transporter ApGLNT1 localizes to the plasma membranes of bacteriocytes and sheath cells [2]. This mature localization of ApGLNT1 differs from the localization patterns we observed in the embryonic bacteriome. While we hypothesized that ApGLNT1 would localize to the plasma membrane of bacteriocytes and sheath cells following cellularization of the embryonic bacteriome (from stage 10 to the end of embryogenesis at stage 20), we only found transient co-localization of ApGLNT1 with F-actin on the inner face of the bacteriocyte plasma membrane at stage 14 (Fig. 4f, f′) and we did not observe a restricted signal surrounding single bacteriocytes in late-stage embryos (Fig. 5b, b′). The apparent transient nature of embryonic bacteriocytes enclosed with ApGLNT1-positive signal at stage 14 leaves us uncertain as to whether the membrane co-localization signals at stage 14 are signals from ApGLNT1 protein in the plasma membrane of bacteriocytes or of signal close to the boundary of bacteriocytes, an uncertainty that can be resolved in future work using higher resolution microscopy of samples co-stained with a plasma membrane-specific marker.

In addition to localizing to the plasma membrane of bacteriocyte and sheath cells in the adult maternal bacteriome, ApGLNT1 localizes as puncta in the cytoplasm of maternal bacteriocytes [2]. During asexual embryogenesis, ApGLNT1 localizes as cytoplasmic puncta throughout development (see especially Figs. 2, 3, 4, and 5b, b′). While Price et al. [2] focused primarily on interpreting the membrane localization of ApGLNT1, the prominent and persistent punctate patterns of cytoplasmic staining in embryos during asexual embryogenesis and in maternal bacteriocytes are remarkable and are interpreted later in this discussion where we suggest that cytoplasmic ApGLNT1 plays a role in nutrient sensing.

In the same way that localization of ApGLNT1 differs in embryonic and maternal bacteriocytes, ApGLNT1 localization differs between embryonic and maternal sheath cells. ApGLNT1 localizes to the plasma membrane of maternal sheath cells, and in contrast, in embryonic sheath cells it localizes as cytoplasmic puncta. Furthermore, the expression of *ApGLNT1* transcript differs between embryonic and maternal sheath cells. *ApGLNT1* mRNA is detected in maternal sheath cells in both first instar nymphs and adults (Fig. 1g, i) [30]; in contrast, we did not detect *ApGLNT1* mRNA in embryonic sheath cells (Additional file 2: Figure S2g, h).

Our finding that ApGLNT1 expression and localization differs between maternal and embryonic bacteriomes demonstrates that bacteriome function is not ontogenetically constant, a pattern consistent with two other recent studies that also demonstrate differences in RNA expression and protein localization in embryonic versus maternal bacteriomes [31, 32]. This lack of equivalency between embryonic and maternal bacteriomes raises intriguing questions about the nutritional contributions of *Buchnera* contained within embryonic bacteriomes during embryogenesis. Which bacteriome(s), the maternal and/or the embryonic, support the extraordinary reproductive capacity of viviparous aphids? Functional characterization and developmental localization of transporters that facilitate transport of *Buchnera* synthesized amino acids and vitamins will facilitate addressing this long-standing question.

### Transovarial glutamine transportation may be mediated by ApGLNT1

There are two membrane barriers that separate maternal hemolymph from developing embryos in asexual viviparous aphids: the follicular epithelium and the embryonic epithelium [33]. The follicular epithelium is the continuous monolayer of epithelial cells that surrounds the germarium and continues along the whole length of the ovariole [9, 34–37]. The embryonic epithelium envelops individual embryos. We interpret the double layer of ApGLNT1 antibody-positive signal to be localized to the outside membrane of the maternal follicular epithelium and the apical membrane of the embryonic epithelium (Figs. 2, 3, 4, 5). As embryos grow within an ovariole, the follicular epithelium stretches so that after stage 7 a double layer of ApGLNT1 signal is difficult to discern. Localization of ApGLNT1 in membranes of two of the cellular barriers that separate maternal hemolymph from embryonic hemolymph indicates that glutamine may be transported from maternal hemolymph to developing oocytes and embryos throughout asexual development.

**Fig. 6:**
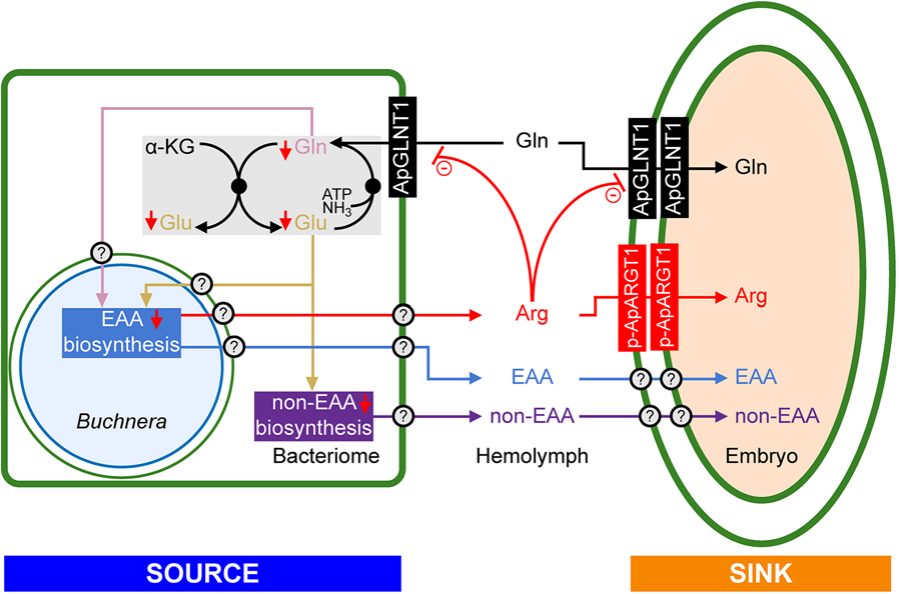
Model for holobiont metabolism regulated by the nutritional demand of the asexual aphid reproductive system. The non-essential amino acid (non-EAA) glutamine (Gln) is transported from hemolymph to the bacteriome and developing embryos by transporter ApGLNT1. Glutamine and glutamate (Glu) are interconverted in the cytoplasm of the maternal bacteriome via the aphid genome-encoded GOGAT cycle (*shaded gray*) [51]. Glutamine and glutamate serve as metabolic precursors for amino acid biosynthesis in the holobiont [1]. We extend Price et al’s [2] regulatory model of amino acid biosynthesis in the maternal bacteriome to include the nutrient demands of embryogenesis such that developing embryos within viviparous aphids act as sinks that consume essential and non-essential amino acids. Arginine (Arg) demand in the embryos will maintain amino acid biosynthesis in the maternal bacteriome. Thus, we propose the localization of an as yet unidentified arginine transporter, putative *A. pisum* Arginine Transporter 1, p-ApARGT1, on the outside membrane of the maternal follicular epithelium and the apical membrane of the embryonic epithelium. The membranes that ApGLNT1 has been shown to localize to are shown with a *thick green line*. These membranes include the bacteriome and follicular epithelial membranes. The symbiosomal membrane and *Buchnera* cell membrane are marked with a *thin green line* and *thin blue line*, respectively. Uncharacterized transporters are indicated by *question marks*

The nutrient provisioning roles of maternal and embryonic bacteriomes during development are far from resolved [38]. Unlike most insects where maturation of oocytes occurs within the ovariole and embryos develop externally, asexual viviparous aphids have telotrophic ovarioles that contain nurse cells (at their terminal tip), developing oocytes and developing embryos that are all bathed in maternal hemolymph. During early development (before stage 4), asexual embryos are provisioned with nutrients by nurse cells via the nutritive cord that directly connects them to the germarium [39, 40]. After dissolution of the nutritive cord, embryos likely receive nutrients directly from the maternal hemolymph. However, following stage 7, when *Buchnera* symbionts invade developing embryos, it is possible that some nutrient provisioning may be compartmentalized within each embryo, with nutrient upgrading and recycling occurring in the embryonic bacteriome. Speculation aside, prior to this study, the role(s) of the maternal versus embryonic symbiosis in nutrient provisioning to mid- and late-stage embryos was undetermined. Here we speculate through localization of *ApGLNT1* mRNA and protein (Figs. 1, 2, 3, 4, 5) that glutamine is transported from maternal hemolymph to developing embryos under conditions of low hemolymph arginine availability throughout development (Fig. 6) [2, 13]. The fact that ApGLNT1 is not found in the membranes of embryonic bacteriocytes also suggests that the mechanisms regulating amino acid biosynthesis in embryonic bacteriocytes differ from those regulating amino acid biosynthesis in maternal bacteriomes [2].

### Cytoplasmic localization of ApGLNT1 suggests that this amino acid transporter may play a role in nutrient sensing

ApGLNT1 is part of the arthropod expansion of mammalian SLC36 family transporters and is related to the mammalian proton-dependent amino acid transporters (PAT1-4) [12, 13]. Notably, both mammalian and *Drosophila* cytosolic SLC36 transporters have been shown to function as intracellular amino acid sensors [41]. Among the metazoans, the major amino acid sensing signaling pathways are the target of rapamycin (TOR) pathway and the general amino acid control non-derepressible 2 (GCN2) pathway [42]. Amino acid transporters play important roles at the top and bottom of both pathways by monitoring both intracellular and extracellular amino acid abundances [43]. In particular, *Drosophila* SLC36 homologs *pathetic* and *CG1139,* and human *SLC36A1* are essential mediators that activate the key factor of Target of Rapamycin Complex 1 (TORC1) kinase in the TOR signaling pathway [41]. Overexpression and mutation of SLC36 gene products affects cell growth through modulating TORC1 [44, 45]. Work with both mammalian and *Drosophila* cytosolic SLC36 transporters suggests that ApGLNT1 may also play a role in nutrient sensing. Alternatively, cytoplasmic localization of ApGLNT1 may result from transporter sequestration in vesicular membranes for later mobilization to the plasma membranes of bacteriocyte and sheath cells, where ApGLNT1 can then function to transport glutamine. That said, the possibility that ApGLNT1 plays both amino acid transport and nutrient sensing roles at the *A. pisum*/*Buchnera* symbiotic interface is intriguing. Resolution of this intrigue will require further experimental work that is outside the scope of the present study.

The intriguing possibility that ApGLNT1 functions during host development both as a nutrient sensor and glutamine transporter raises questions about how symbiotic function changes during development. Recent work comparing protein expression patterns in embryonic and maternal bacteriocytes revealed that protein expression differs between the embryonic and post-embryonic developmental stages, especially with respect to proteins related to the biosynthesis of essential amino acids [31], a result that is consistent with other studies that also found ontogenetic shifts in gene expression of genes central to the aphid–*Buchnera* nutritional association [32, 46, 47]. Changes in symbiotic function in response to nutrient demand or environmental cues are also integrated into host developmental programs in the human-gut microbiome [48], the squid-vibrio symbiosis [49], and the symbiosis of the salamander *Ambystoma maculatum* with its algal symboint, *Oophila amblystomatis* [50]. In-depth investigation of the cellular function of symbiont-biased genes during host development may facilitate elucidation of how symbiotic partners are integrated to generate the holobiont or, more particularly, how vertically transmitted symbionts are integrated into the developmental programs of their hosts.

## Conclusions

That ApGLNT1 localizes to the *A. pisum* follicular epithelium suggests transport of glutamine from maternal hemolymph to embryos throughout asexual embryogenesis. Consistent with previous work, we show that the maternal and embryonic bacteriomes are not equivalent [31, 32, 46] and venture that the extraordinary reproductive capacity of asexually reproducing aphids is largely supported by the maternal symbiosis. Remarkably, our work suggests that maternal bacteriocytes do not synthesize *ApGLNT1* transcripts, but rather are provisioned with ApGLNT1 by neighboring sheath cells. Finally, the subcellular localization of ApGLNT1 in maternal and embryonic sheath and bacteriocyte cells informs our proposal that in addition to its role in nutrient transport, ApGLNT1 likely plays an important role in nutrient sensing at the *A. pisum*/*Buchnera* symbiotic interface.

## Additional files

10.1186/s13227-015-0038-y Western blot analysis of recombinant *A. pisum* ApGLNT1 in yeast cell membranes. Western blot analysis of total membranes (20 mg membrane protein/lane) from *S. cerevisiae* strain 22Δ8AA [23] transformed with empty pDR195 expression vector (control, lane 1) and cells expressing ApGLNT1 (ApGLNT1, lane 2). Yeast membrane proteins were separated on a 12.5 % polyacrylamide gel, transferred to nitrocellulose and probed with primary anti-ApGLNT1 antibody and a fluorescently labeled secondary antibody. Arrow indicates presence of an ApGLNT1 antibody immunoreactive band, which is only present in cells expressing ApGLNT1 (ApGLNT1, lane 2). The calculated relative molecular mass (*M*_r_) of ApGLNT1 is 55.1 kDa, but as analyzed by SDS-PAGE the protein has an apparent *M*_r_ of 42 kDa. The anomalous migration of ApGLNT1 on SDS-PAGE gels is a due to the highly hydrophobic nature of the protein, and is consistent with other hydrophobic transmembrane transporters [52].
10.1186/s13227-015-0038-y Transcripts of *ApGLNT1* localized to the maternal follicular epithelium in the asexual embryos. Dissected ovarioles were hybridized with antisense (a-i) and sense riboprobes (j-r). Anterior region of egg chambers is to the left and dorsal is to the top. After katatrepsis, the head of germband reverse the position to the anterior of the egg chambers (i, r). Antisense positive signals mark with closed arrowheads in the follicular epithelium and arrow in the central syncytium. Scale bars: 20 μm. Abbreviation: b, endosymbiotic bacteria *Buchnera*; bc, bacteriocyte; gc, germ cells; gm, germarium; hd, head; st, stage.
10.1186/s13227-015-0038-y Cytoplasmic localization of ApGLNT1. Embryonic bacteriocytes movie in a stage 13 embryo constructed from serial confocal sections. Green, red, and blue signal are ApGLNT1-positive signal, F-actin and DNA.

## Abbreviations

AAAP: amino acid/auxin permease family transporters
ApGLNT1: *A. pisum* glutamine transporter 1
BCIP: 5-bromo-4-chloro-3-indolyl phosphate
DAPI: 4′,6-diamidino-2-phenylindole
DIG: digoxigenin
GCN2: general amino acid control non-derepressible 2
NBT: nitroblue tetrazolium
NCBI: National Center for Biotechnology Information
PATs: proton-dependent amino acid transporters
PBS: phosphate-buffered saline
phalloidin–TRITC: phalloidin–tetramethylrhodamine B isothiocyanate
SC: synthetic complete medium
SLC36: solute carrier family 36
SLC36A1: solute carrier family 36 member 1
TOR: target of rapamycin
TORC1: target of rapamycin complex 1 kinase
