# Supplementary figures and images for: Ontogenetic differences in localization of glutamine transporter ApGLNT1 in the pea aphid demonstrate that mechanisms of host/symbiont integration are not similar in the maternal versus embryonic bacteriome
Source: EvoDevo. 2016 Jan 11;7:1. doi: 10.1186/s13227-015-0038-y (PMC4709974; doi:10.1186/s13227-015-0038-y)

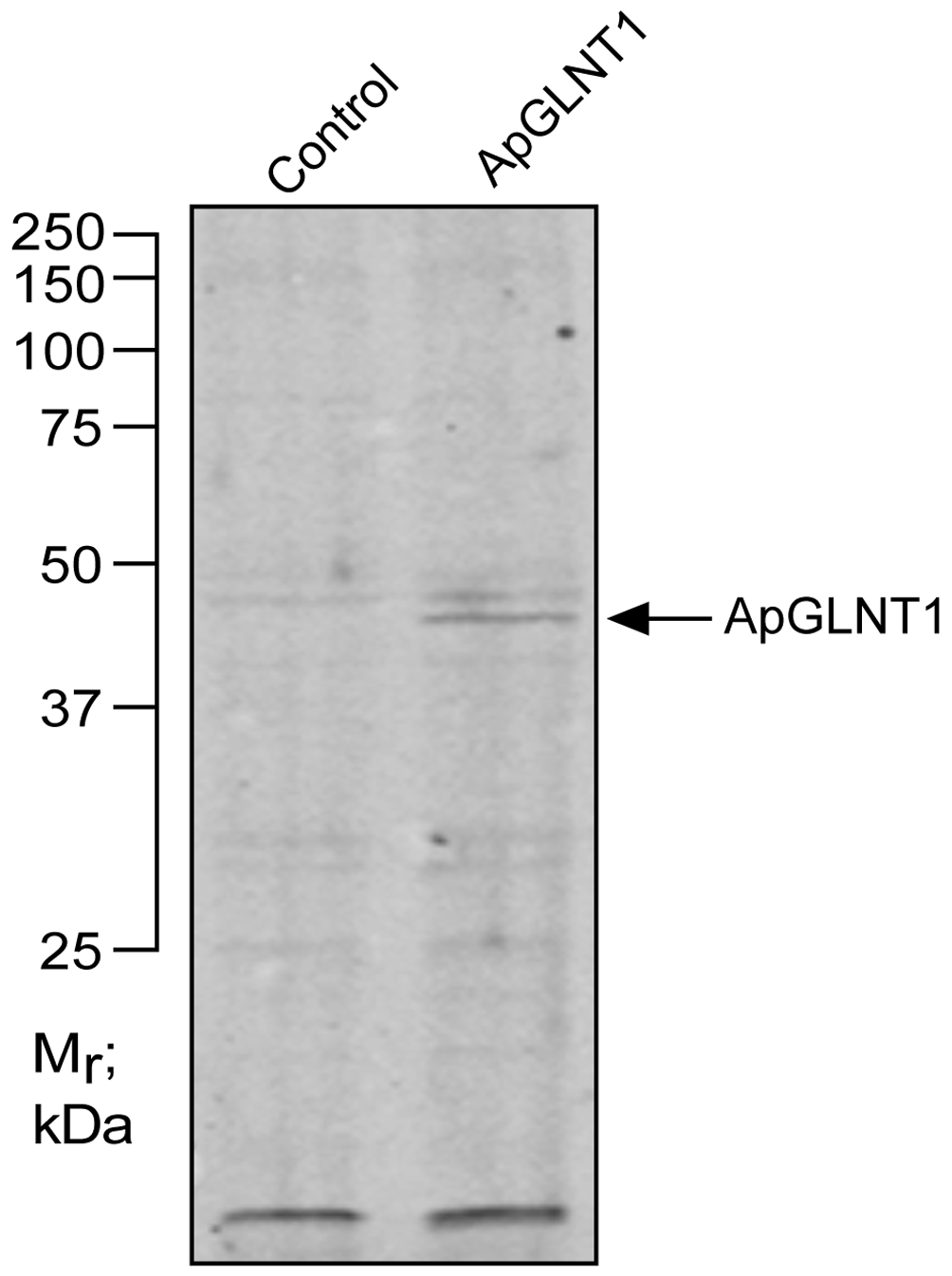

Supplement: Supplementary file 1 — 10.1186/s13227-015-0038-y Western blot analysis of recombinant A. pisum ApGLNT1 in yeast cell membranes. Western blot analysis of total membranes (20 mg membrane protein/lane) from S. cerevisiae strain 22Δ8AA [23] transformed with empty pDR195 expression vector (control, lane 1) and cells expressing ApGLNT1 (ApGLNT1, lane 2). Yeast membrane proteins were separated on a 12.5 % polyacrylamide gel, transferred to nitrocellulose and probed with primary anti-ApGLNT1 antibody and a fluorescently labeled secondary antibody. Arrow indicates presence of an ApGLNT1 antibody immunoreactive band, which is only present in cells expressing ApGLNT1 (ApGLNT1, lane 2). The calculated relative molecular mass (M r) of ApGLNT1 is 55.1 kDa, but as analyzed by SDS-PAGE the protein has an apparent M r of 42 kDa. The anomalous migration of ApGLNT1 on SDS-PAGE gels is a due to the highly hydrophobic nature of the protein, and is consistent with other hydrophobic transmembrane transporters [52]. [file 13227_2015_38_MOESM1_ESM.tif]

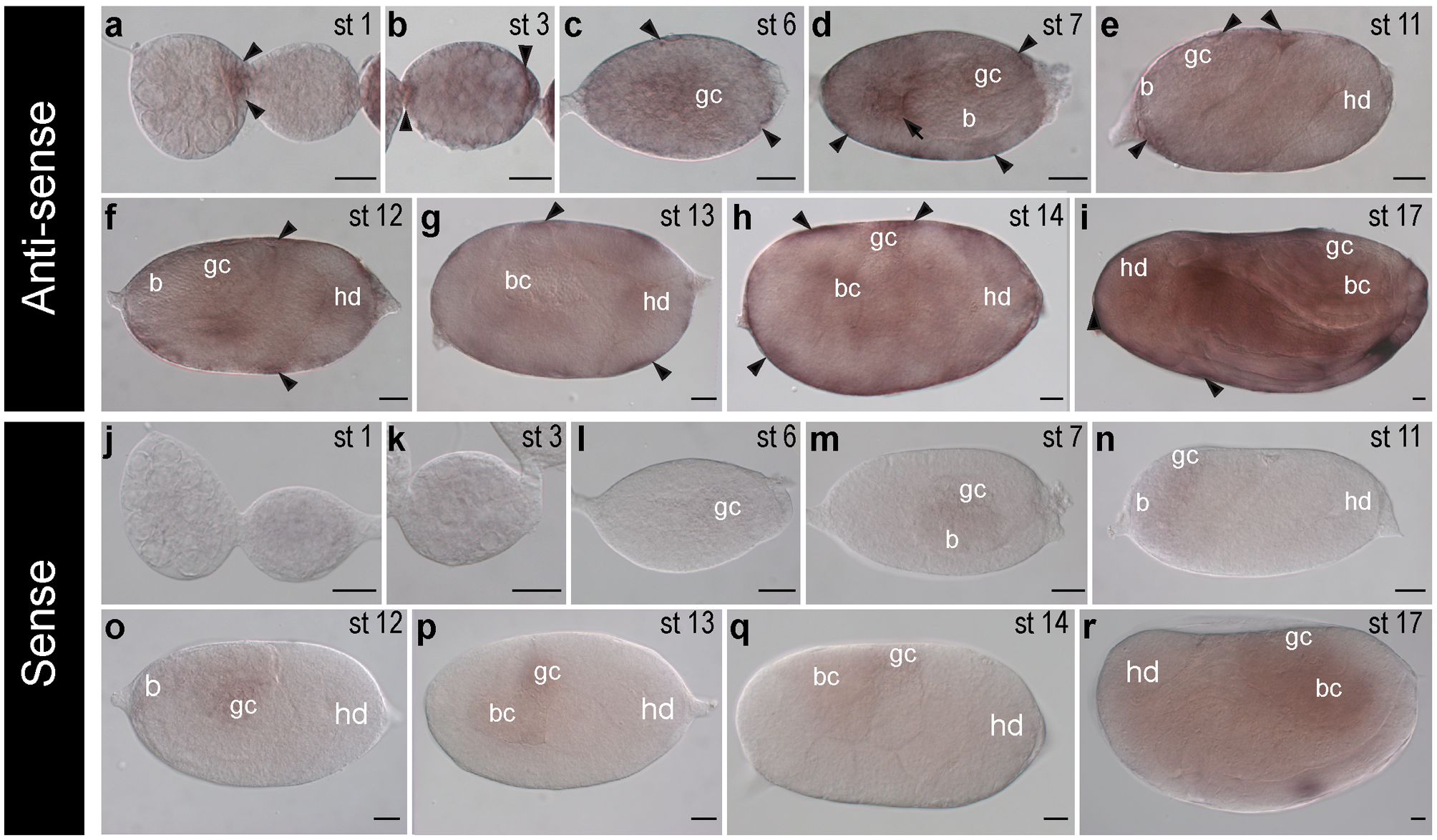

Supplement: Supplementary file 2 — 10.1186/s13227-015-0038-y Transcripts of ApGLNT1 localized to the maternal follicular epithelium in the asexual embryos. Dissected ovarioles were hybridized with antisense (a-i) and sense riboprobes (j-r). Anterior region of egg chambers is to the left and dorsal is to the top. After katatrepsis, the head of germband reverse the position to the anterior of the egg chambers (i, r). Antisense positive signals mark with closed arrowheads in the follicular epithelium and arrow in the central syncytium. Scale bars: 20 μm. Abbreviation: b, endosymbiotic bacteria Buchnera; bc, bacteriocyte; gc, germ cells; gm, germarium; hd, head; st, stage. [file 13227_2015_38_MOESM2_ESM.tif]
